# COVID-19, State of the Adult and Pediatric Heart: From Myocardial Injury to Cardiac Effect of Potential Therapeutic Intervention

**DOI:** 10.3389/fcvm.2020.00140

**Published:** 2020-07-14

**Authors:** Dominga Iacobazzi, Mai Baquedano, Paolo Madeddu, Massimo Caputo

**Affiliations:** Bristol Heart Institute, Translational Health Sciences, Bristol Royal Infirmary, University of Bristol, Bristol, United Kingdom

**Keywords:** COVID-19, cardiovascular disease, congenital heart defect, pediatric, cell therapy

## Abstract

While the COVID-19 pandemic continues to spread rapidly, resulting in considerable morbidity and mortality worldwide, multiple efforts are being made by the international scientific community to understand the pathogenesis of the viral infection and its clinical outcome. Older age and comorbidities have consistently been reported as risk factors for unfavorable prognosis, with cardiovascular disease accounting for up to 10 % of comorbid conditions among the infected patients. An understanding of the mechanism underlying the effect of this infection on patients with cardiovascular disease is essential to manage and improve clinical strategies against the disease in that population. In this review, we summarize the impact of COVID-19 on patients with underlying cardiovascular conditions and the cardiac implications of known and emerging therapeutic strategies. Our future effort will aim to further elucidate how the type and severity of the cardiac disease, with particular regard to Congenital Heart Disease, influences the prognosis and the outcome of the viral infection.

## Introduction

COVID-19 is a severe acute respiratory illness caused by a new coronavirus (SARS-CoV-2), which is recognized as the third human pathogen causing a severe respiratory syndrome after SARS-CoV and Middle East Respiratory Syndrome (MERS)-CoV1 (1, 2).

Although most infected people only show mild-to-moderate respiratory symptoms or nothing at all, up to 10% of infected individuals manifest the complete acute respiratory distress syndrome (ARDS) of COVID-19 (3, 4). Pre-existing medical conditions and comorbidities, along with the advanced age, are considered key factors for the outcome of the disease (3, 5, 6).

## Cardiovascular Involvement in the COVID-19 Pandemic

Multiple studies have shown that patients with underlying cardiovascular disease (CVD) such as hypertension and coronary artery disease are more likely to suffer from a severe COVID-19 infection that requires ICU care (3, 7, 8). In addition, data from all past influenza pandemics, (i.e., the 1918 flu, SARS, and MERS) revealed that cardiovascular events surpassed all other causes of mortality, including superimposed pneumonia (9–11).

As a matter of fact, there seems to be a particular interplay between the 2019 novel coronavirus and the cardiovascular system. The already increased metabolic demand and the reduced/impaired cardiovascular reserve in CVD patients could explain the association between the two pathological conditions (12).

The overwhelming immune inflammatory response and the subsequent cytokine storm that hits the heart as the delayed consequence of the SARS-CoV-2 infection contributes to worsening the precarious cardiac status via the mechanism of inflammation-induced heart failure. This includes endothelial dysfunction due to imbalance between ROS production and NO reduction, left ventricular remodeling, and fibrosis by differentiation of fibroblasts into myofibroblasts following transforming growth factor beta (TGFβ) secretion by monocytes (13, 14).

In addition, the heart might indirectly suffer from pulmonary injury caused by direct viral infection of lung epithelia via ACE2, which causes pulmonary damage and hypoxemia and, in turn, may lead to further end-organ dysfunction, such as oxidative stress-induced injury in the heart (3, 15).

A direct viral infection of the cardiomyocytes is also plausible due to the presence of ACE2 in the heart. Previous SARS epidemic post-mortem analysis showed the presence of viral genome in heart biopsies; however, this evidence has not yet been demonstrated in SARS-CoV-2 infection (16).

It is likely that, to different extents, each of these mechanisms, or a combination of them, plays a role in the cardiac injury that manifests in the late hyper-inflammatory phase of the viral infection. The high levels of cytokines detected during the advanced stage of the illness (IL-6, IL-2, IL-7, TNF-α, and interferon-γ) correlates with elevated levels of biomarkers of cardiac injury, both of which increases are associated with worse prognosis (5–7, 17–19). For example, the elevation of troponin I (TnI) and brain-type natriuretic peptide (BNP) biomarkers was found to be a predictor of in-hospital death (5, 7, 8, 20, 21). Elevated inflammatory markers also correlate with electrocardiographic abnormalities, arrhythmias, and blood pressure disorders, whose incidence has been reported to be significantly higher among those requiring ICU care (44.4 vs. 6.9%) compared with those treated in non-ICU beds (18, 22). Table 1 summarizes the cardiovascular comorbidity and cardiac injury in COVID-19 patients.

**Table 1:** Characteristics of COVID-19 patients with cardiovascular disease.

**References**	**Patients (*n*)**	**Age**	**Cardiovascular comorbidity**	**CHD history**	**Cardiac injury**	**Death**
Yang et al. (3)	52	30–80 years	Chronic cardiac disease (5 pt)	NA	Acute cardiac injury (high Troponin I)	Yes (3 pt)
Zhou et al. (7)	191	46–67 years	Hypertension 58 pt Coronary artery disease (15 pt)	No	Heart failure, acute cardiac injury	Yes (39 pt)
Arentz et al. (8)	21	43–92 years	Congestive heart failure (9 pt)	No	Cardiomyopathy	NA
Sabatino et al. (23)	76	1–52 years	Hypertension (5 pt) Prev. cardio-embolic stroke (2 pt)	Yes (9 pt)	Heart failure, arrhythmias, myocardial injury	No

## COVID-19 and Adult Congenital Heart Disease

To date, there is no evidence on COVID-19 complications in patients with congenital health disease (CHD). To the best of our knowledge, only one multi-center, observational Italian study has assessed, through a nationwide survey, the clinical characteristics and outcomes in patients with CHD affected by COVID-19 (23). Out of the 76 patients included in the study, nine patients (seven adults, two children) had a history of CHD, including atrial septal defect, ventricular septal defect, tetralogy of fallot, coarctation of aorta, and pulmonary atresia. Cardiovascular complications, in the form of heart failure, arrhythmias, and myocardial injury, were mainly observed in the CHD-COVID-19^+^ group; however, no critical respiratory outcome and no virtual death were observed. Although the authors concluded that the overall mild clinical courses observed in the current panorama of literature reporting an association between cardiovascular risk factor and higher fatality rate in COVID-19 patients were rather reassuring, more randomized trials involving larger populations are needed.

In the absence of wider and conclusive data, the Adult Congenital Heart Association and the British Congenital Cardiac Association have included this patient population among the at-risk group, given the profound impact of the virus on the heart of CVD patients. In particular, complex CHDs, such as cyanotic heart defects, single ventricle, pulmonary atresia, and truncus arteriosus, have been listed as higher risk conditions on the basis of decreased functional reserve (24, 25). Therefore, it is strongly advised that these patients take great care in preventing infection, adopting social distancing measures, frequent handwashing, and proper use of personal protective equipment. Diagnosis of suspicious cases in CHD individuals is similar to in the general population; however, further diagnostic methods, such as chest CT, might be warranted in addition to the canonical nasopharyngeal swab and RT-PCR tests (26). Furthermore, the evaluation of cardiac injury markers could be important for the management of COVID-19-positive CHD patients showing signs of acute coronary syndrome (i.e., chest pain), arrhythmias, and myocardial injury (i.e., abnormal ECG), for which the use of supplemental oxygen might not be sufficient. The opportune use of mechanical ventilation, fluid resuscitation, and extracorporeal membrane oxygenation could be of additional support in such cases, as it has proved to be successful in critically ill patients (3, 27).

## COVID-19 and the Pediatric CHD Population

No studies have reported, as yet, on the correlation between COVID-19 and children with CHD. The pediatric population seems, overall, to be less vulnerable to COVID-19 than adults. A restrained cytokine storm as a result of a not fully developed adaptive immune response, together with less functional ACE2, which makes SARS Cov2 less infectious, has been suggested as the main mechanism underlying the reduced susceptibility of children (28). Nevertheless, young children and infants appear to be more susceptible to developing severe disease than older children (4, 29). A case report from China described the case of a COVID-19-positive 55-day-old infant with pneumonia, liver injury, and heart damage, as revealed by abnormal myocardial zymogram and occasional arrhythmias (30). In another small case series of critically ill patients with COVID-19, the case of a 13-month-old child who developed heart failure, together with multiple organ failure was reported (31). Multiorgan failure was also the cause of the death of a 10-month-old child with a history of intussusception (32).

As seen in the adult population, comorbidity is also a critical factor for SARS-CoV-2 infection, and children with underlying disease are more likely to be more susceptible to COVID-19. A retrospective study carried out in China involving 25 pediatric patients showed that the most aggressive COVID-19-related infection was detected in two patients previously operated for CHD (33). In another retrospective study analyzing the clinical, laboratory, and chest CT features of 20 pediatric inpatients with COVID-19 in Wuhan, it was reported that 7 out of 20 hospitalized patients had a previous history of congenital or acquired disease, with two cases having a history of atrial septal defect surgery. However, the authors did not report whether the pre-existing conditions resulted in a worse or longer prognosis, which is a limitation of the study (34). Interestingly, the laboratory findings showed that in addition to the increased levels of common inflammatory markers and abnormal CT chest also reported in adults, almost all the cases (16 out of 20, 80%) presented elevated levels of procalcitonin (PCT), which is not common in adult patients and, therefore, worth further investigation. PCT is a diagnostic factor more specific for bacterial infections, which indeed was found in half the analyzed patients, suggesting that routine antibacterial treatment might be considered in such pediatric patients (35).

In the absence of any available data on COVID-19 in children with CHD, this population has been categorized, as in adults, as a group that is particularly vulnerable to becoming unwell with respiratory infections, including COVID-19 (24). Therefore, the same particular care must be taken for this population, especially for those affected by cyanotic heart conditions (oxygen saturation <85% at rest), which are likely to worsen in a hypoxemic pulmonary circulation setting.

Based on a literature review of published and unpublished data, a team of experts at Alder Hey Children's Hospital, Liverpool, has set up some guidance for the clinical management of children admitted to hospital with proven COVID-19 (36).

As a guideline for the treatment of hypoxic children developing respiratory failure and requiring respiratory support, the authors recommend that patients receive low flow nasal cannula (LFNC) oxygen rather than high flow nasal cannulae (HFNC) in order to reduce droplet spread of the virus. However, if children are hypoxic despite LFNC, then HFNC can be tried. In addition, although there is no evidence in the literature regarding blood gases and these should not be routinely used, their use is justified in children who, despite administration of HFNC, require further respiratory support.

Clinical studies enrolling the pediatric CHD population are needed in order to collect data to better understand outcomes in this population. In the field of cardiac surgery, few surveys have been developed in the UK in order to explore how the surgical case mix and to link this information with a national database with perioperative outcomes as a primary endpoint (37). Meanwhile, the patient waiting list has changed during this pandemic both in adults and in congenital cardiac surgery.

Concerning data have emerged in the last few weeks regarding an apparent rise in the number of children of all ages presenting with a multisystem inflammatory state, typical of Kawasaki disease (KD), requiring intensive care across the UK and several other countries (38).

**Figure 1:**
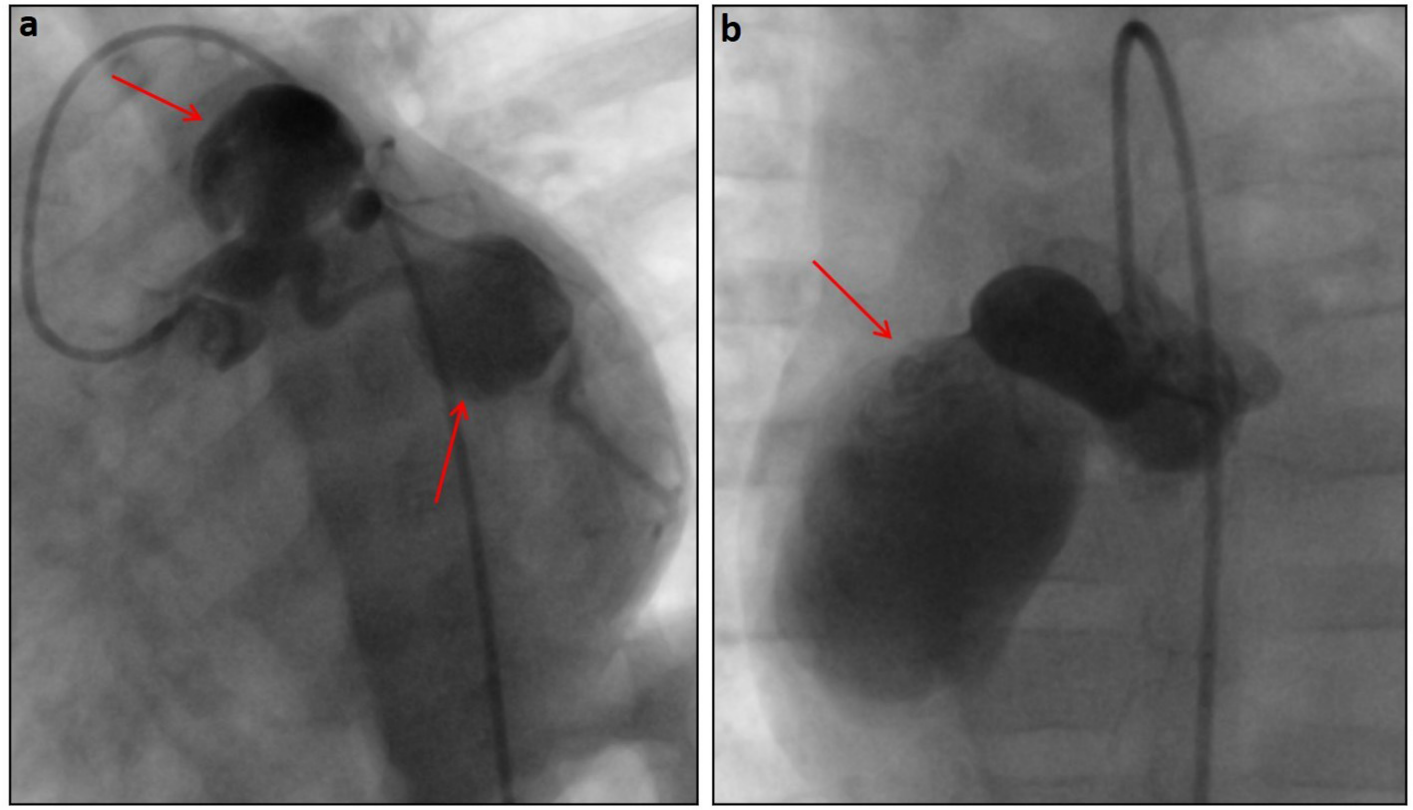
Angiographic pictures of very large saccular aneurysms (red arrows) in the left coronary **(a)** and right coronary **(b)** systems in a 6-month-old patient diagnosed with Kawasaki disease.

KD is a rare acute pediatric vasculitis, with coronary artery aneurism (CAA) as its main complication (Figure 1). One pronounced feature of KD is persistent fever, accompanied with exanthema, lymphadenopathy, and changes to mucosae (39). Prompt diagnosis and treatment with intravenous immunoglobulin (IVIG) results in complete recovery of patients in most cases, preventing possible CAA complications (40).

Some of the children that, in the present circumstance, developed KD-like symptoms had tested positive for COVID-19, whilst others had not, suggesting that the critical systemic toxic shock symptoms could be related to another unidentified infectious pathogen. However, even though the relationship of KD to COVID-19 is not yet defined, there is growing concern that SARS-CoV-2 infection might be linked with the concomitant inflammatory syndrome that is affecting some young children. Congruent with this notion are the data from an Italian cohort study, reporting the case of ten children with Kawasaki-like disease, some of which required fluid resuscitation and inotropic support, and all of whom tested positive for SARS-CoV-2 either by swab or serology test (41).

An association between the novel virus and KD in children is further supported by a French study describing a case series of major systemic inflammation and acute myocarditis following SARS-CoV-2 infection in 20 critically ill children (42). In all of the patients, a remarkably high rate of IgG and IgA was detected, suggesting a post-viral immunological reaction impacting the myocardium. The significant reduction of inflammatory biomarkers and the cardiac function improvement following IVIG further reinforce the hypothesis of a SARS-CoV-2 post-infective disease. The cardiac involvement of pediatric inflammatory multisystem syndrome associated with SARS-CoV-2 was also reported by another single-center retrospective study conducted at Birmingham Children's Hospital (43). All of the pediatric patients presented impaired left ventricular function, valve regurgitation, and/or coronary artery involvement, which reflects a significantly higher cardiac involvement compared to the Italian and French series.

Although all of these studies suggest that the emerging severe multisystem inflammatory disease might be one of the unknown clinical post-infective complications of the SARS-CoV2 infection, long-term follow-up and further research are needed to understand the immunopathology and the potential mechanistic link between COVID-19 and Kawasaki disease.

Table 2 summarizes the characteristics of the studies focused on cardiovascular involvement in COVID-19-affected children.

**Table 2:** Review of COVID-19 pediatric patients and cardiovascular involvement.

**References**	**Patients (*n*)**	**Age**	**CHD history**	**Cardiovascular symptoms**	**Cardiovascular tests**	**Therapy**
Cui et al. (30)	1	55 days	No	Tachycardia	Troponin	i.v sodium creatine phosphate and oxygen
Sun et al. (31)	8	2 months−15 years	No	Heart failure (1pt)	Non-specific for CV disease	Plasmapheresis and oxygen
Lu et al. (32)	1	10 months	No	Heart failure	NA	NA
Zheng et al. (33)	25	3 months−14 years	Yes (2 pt)	No	Cardiac enzymes	Antiretroviral agents, corticosteroids, i.v immunoglobulin
Xia et al. (34)	20	1 day−14 years	Yes (2 pt)	Abnormal EGC	Procalcitonin	NA
Verdoni et al. (41)	29	3–7 years	No	Kawasaki disease	Cardiac enzymes	i.v immunoglobulin, aspirin, methylprednisolone
Grimaud et al. (42)	20	3–15 years	No	Kawasaki disease	Cardiac enzymes	corticosteroids, i.v immunoglobulin
Ramcharan et al. (43)	15	6–11 years	No	Kawasaki disease, abnormal EGC, LV dysfunction, atrioventricular valve regurgitation	Cardiac enzymes	i.v immunoglobulin, aspirin, methylprednisolone

## Therapeutic Approach and the Cardiac Concern

Since the spread of the pandemic in January 2020, there has been a rapidly growing number of clinical trials; however, to date, no definitive therapies, other than supportive care, are available. An extensive review of the therapeutic agents under investigation for the treatment of COVID-19 is beyond the scope of this review. We, therefore, intend to focus on the cardiac involvement of the therapeutics that have been employed or are under investigation as options for COVID-19 treatment (Table 3).

**Table 3:** Cardiac involvement of medications used in COVID-19 patients.

**Medication**	**Cardiac concern and recommendation**
Antivirals	No proven efficacy on COVID-19 patients. Some of these agents can cause cardiac insufficiency, arrhythmias, or other cardiovascular disorders.
Antimalarial	Limited human data to support effectiveness in humans. Some of these medications can prolong the QT interval; this should be taken into consideration, especially in patients on other QT-prolonging agents.
Corticosteroids	Can cause or exacerbate existing lymphocytopenia. Not recommended in the first phase of the infection; can be favorable in the later hyperinflammatory stage.
ACEi/ARB	Theoretical concern because of potential upregulation of ACE-2. However, no data support interaction with the disease. Suggest continuing the medication.

Antivirals, such as Lopinavir and Ritonavir, were reported to have the potential to treat SARS infections; however, no efficacy in COVID-19 patients has been proven so far (44, 45). In addition, many antiviral drugs are known to cause cardiac insufficiency, arrhythmia, or other cardiovascular disorders (46). Cardiovascular toxicity (conduction abnormalities and long QT syndrome) is also associated with the antimalarial agents chloroquine and hydroxychloroquine, which have been proposed as off-label agents to treat SARS-CoV-2 infection after proving effective against SARS CoV-2 *in vitro* (47, 48). Preliminary results from clinical trials on COVID-19 patients remain not fully convincing, and further evaluation is needed (49). Therefore, the use of these agents during treatment of COVID-19 conditions must be carefully considered and the risk of cardiac toxicity closely monitored in patients with pre-existing cardiovascular conditions.

The use of corticosteroids, although not recommended in the first phase of the infection due to the side effect of worsening the lymphocytopenia that manifests in the acute disease progression, can be justified in the later hyperinflammatory stage. Furthermore, lower incidence of myocardial infarction among patients with pneumonia was associated with corticosteroid treatment, and glucocorticoid treatment proved to be lifesaving for a 37-year-old man with fulminant myocarditis (50).

There has been a tremendous amount of speculation surrounding the potential adverse effect of angiotensin-converting enzyme inhibitors (ACEi) and angiotensin receptor blockers (ARS) in CVD patients with COVID-19. In animal models, these drugs increased cardiac ACE2 expression after chronic treatment (51). Though this effect, on the one side, would increase the number of receptors available for SARS-CoV-2 binding, on the other side, it might be protective against lung and heart injury as a consequence of the reduction of angiotensin II levels and increased production of the tissue protector Angiotensin 1–7. In fact, following SARS-CoV-2 binding to ACE2, the latter is internalized and degraded; therefore, COVID-19 is characterized by ACE2 depletion and endothelial dysfunction. While more studies are needed to elucidate the effect of angiotensin-converting enzyme inhibitors and angiotensin receptor blockers in COVID-19 CVD patients, the American Heart Association and the European Society of Cardiology have recommended the continuation of RAAS inhibitors for patients currently taking them for indications for which these agents are known to be beneficial (52, 53).

## Advanced Treatment: Cell Therapy

Among the many clinical trials currently ongoing (over 80 at the time of this review) that aim to find a therapeutic solution to the COVID-19 pandemic, there are some emerging studies involving cell therapy. Such trials are motivated by the rationale that cells with immunomodulatory properties, along with regenerative capacity, might be beneficial in treating the infection. Based on these peculiar features, cell-based therapy has been incorporated into treatment plans for a number of diseases, including pulmonary and cardiovascular diseases, and immune-mediated inflammatory conditions, such as graft-vs.-host disease (GVHD) and systemic lupus erythematosus (54–56).

Mesenchymal stem cells, somatic progenitor cells, appear to be an attractive candidate for a cell-based therapeutic approach in COVID-19 owing to their powerful immunomodulatory ability, which might be crucial in preventing or attenuating the deleterious cytokine storm to the lung and, as a result, to multiple end-organs (57).

**Figure 2:**
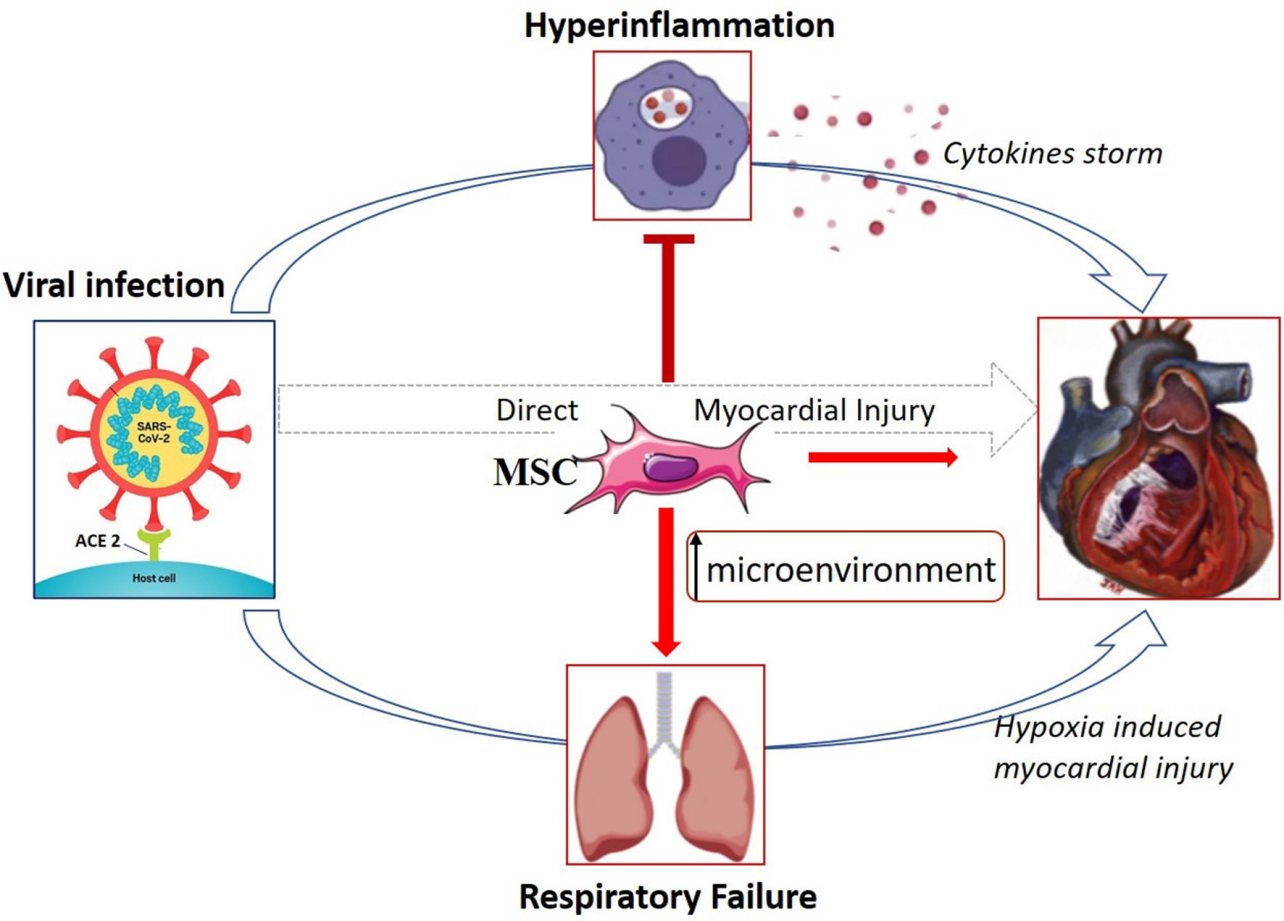
Possible mechanism of THE therapeutic effect of MSCs in COVID-19 treatment. MSCs might exert their beneficial effect through immunomodulatory capacity by interacting with immune cells and, in turn, avoid the cytokine storm that causes lung, and multiple organ injury. Furthermore, owing to their regenerative properties, they could improve the lung microenvironment through the release of trophic factors that protect epithelial cells and improve alveolar cell function. A direct effect of MSCs on the heart in this setting is uncertain. Adapted from References (15, 27).

In addition, their differentiation and regenerative capacity, through the release of trophic factors (IL-10, VEGF, FGF, etc.), could improve the lung microenvironment, where they accumulate after intravenous infusion, and exert their effect by protecting epithelial cells, preventing pulmonary fibrosis, and improving alveolar cell function (54, 58). Importantly, MSCs do not express ACE2 on their cellular surface, indicating that these cells are free from COVID-19 infection (59) (Figure 2). It is, however, not clear if this cell type, as well as other low ACE2 expressing cells, can be infected through other entry receptors.

Two recent Chinese studies have investigated the effect of intravenous administration of clinical-grade human MSCs into patients with COVID-19. In the study by Dr. Zhao and collaborators, the seven patients positive for SARS-CoV-2 with pneumonia recovered from all the symptoms (high fever, shortness of breath, and low oxygen saturation) by 2–4 days after receiving the MSC infusion. In addition, chest CT imaging demonstrated that chest pneumonia infiltration significantly subsided, and also pro-inflammatory cytokines reduced (59).

The second study is a case report of a critically ill 65-year old Chinese woman infected with COVID-19 whose conditions improved after the infusion of allogeneic umbilical cord-derived MSCs. Despite the significant clinical outcome and good tolerance, conclusions cannot be drawn on the base of a single case, especially given that the MSC treatment was superimposed on other therapies, including antivirals, immunoglobulin, and corticosteroids (60).

Whether injected MSCs would have a beneficial effect on an injured heart as a consequence of the viral infection is unclear. However, it is reasonable to hypothesize that, given the modality of systemic administration, the majority of MSCs would accumulate in the pulmonary vascular bed where they are cleared within 24–48 h, as demonstrated by tracking studies using labeled MSCs. Therefore, the amount of cells reaching the heart might not be significant enough to support myocardial regeneration (61).

Nevertheless, concerns regarding the safety of these infusions must not be underestimated, as the potential thrombogenic risk associated with their intravenous administration cannot be excluded. Activation of coagulation was reported in several transplanted patients and could be linked to the expression of tissue factor (TF) on MSCs that, when in contact with blood, initiates coagulation (62). Embolus formation from transplanted MSCs in the lungs of small and large animals has been described, as has lethal pulmonary thromboembolism following the administration of adipose-derived MSCs in some patients (62, 63).

The vessel blockage could provide further hypoxic stress to the lung and related tissues, especially those with high-oxygen consumption and nutritional demand, such as the myocardium, which can ultimately result in additional cardiac damage.

Despite the paucity of pre-clinical and clinical data showing an effective benefit of MSCs on COVID-19 patients, the number of MSC-based clinical trials aimed at exploring the therapeutic potential of cell treatment for SARS-CoV-2-infected patients is rapidly increasing.

Researchers and clinicians in the United States have urged the US administration and COVID-19 task force to minimize regulatory burden by all agencies so that umbilical cord-derived MSCs can be considered for compassionate use in critically ill COVID 19 patients who are not responding to conventional therapies in order to reduce morbidity and mortality in the United States (64).

A team of scientists led by Dr. Ricordi at the Cell Transplant Center, University of Miami Miller School of Medicine, has recently been granted immediate US Food and Drug Administration (FDA) authorization for a 24-patient clinical trial to test the safety and exploratory efficacy of umbilical cord-derived mesenchymal stem cells (UC-MSCs) to prevent the life-threatening lung inflammation that accompanies severe cases of COVID-19 (65).

Based on the same principle as expanded MSCs, cardiosphere-derived cells (CDCs) have been proposed for the treatment of critically ill COVID-19 patients (15). CDCs are stromal progenitor cells isolated and grown from human heart tissue, with proven *in vivo* myocardial protection, anti-inflammatory effects, and immunomodulatory properties (15, 66).

Professor Marban, a pioneer of the CDC technique, has recently proposed the (CS)^3^ trial, aimed at enrolling critically ill patients with pneumonia and signs of lymphocytopenia and cytokine storm and evaluating the effect on cardiac and immune function, as well as the outcome on mortality, length of stay in ICU, and duration of ventilatory support.

## Perspectives

Despite the promise and excitement surrounding cell therapy in COVID-19 disease, precautions must be taken to not create false hopes, as we witnessed in the past when stem cells were sold directly to patients as “cures” without systematic clinical trials to test for safety and effectiveness.

Rigorous clinical trials are needed, which must be designed to strict standards, including detailed information as to inclusion and exclusion criteria, comorbidities, dose and source of cell, timing of administration relative to onset of disease, recognized endpoints, and follow up.

The same caution must accompany the results and interpretation of all trials that are being conducted worldwide to avoid declaration of unproven efficacy such as where drugs have been presented as “game changers” despite showing only tentatively positive results.

SARS-CoV-2 is a new pathogen, and its long-term effects are unknown. Organ complications might persist even after the resolution of acute illness. The previous SARS epidemic is informative as to the possible long-term consequences of the disease. Long-term follow-up studies showed that, 12 years after contracting the virus, patients who recovered from SARS-CoV in 2003 were affected by cardiovascular abnormality, serum metabolic disorders, and pulmonary fibrosis (67, 68).

In the uncertain scenario of the COVID-19 pandemic, the mobilization of the scientific and medical community has been immensely inspiring. It is important that speculation and political pressure to accelerate approvals for treatments that are not supported by sound science do not take over the communal research effort toward understanding in detail the biology of SARS-CoV-2 and developing an effective vaccine, together with new therapies with proved safety and efficacy.

More studies are also needed to evaluate the effect of SARS-CoV-2 and potential therapies on populations classified as risk groups but with no clear evidence on the consequence of the COVID-19 disease. Adult and pediatric patients with congenital heart defects are among these groups. As a team involved in CHD research and surgery, we are aiming at analyzing blood and urine samples that we have been collecting from patients undergoing routine corrective heart surgery and correlating the presence of COVID-19, whenever ascertained, with effects on cardiac biomarkers, function, and clinical outcome. The study aims to further elucidate whether the type and severity of CHD have an impact on the expression of specific markers and clinical outcomes in COVID-19 patients and whether these could be predictive factors of the progression of the disease. This knowledge would provide effective tools for the management of specific CHD conditions in the presence of COVID-19 and other hyperinflammatory syndromes of viral or non-viral etiology that might affect the heart and vasculature.

## Author Contributions

DI drafted the manuscript. MB participated in data screening. PM and MC edited and approved the final version. All authors contributed to the article and approved the submitted version.

## Conflict of Interest

The authors declare that the research was conducted in the absence of any commercial or financial relationships that could be construed as a potential conflict of interest.
